# SARAH Domain-Mediated MST2-RASSF Dimeric Interactions

**DOI:** 10.1371/journal.pcbi.1005051

**Published:** 2016-10-07

**Authors:** Goar Sánchez-Sanz, Bartłomiej Tywoniuk, David Matallanas, David Romano, Lan K. Nguyen, Boris N. Kholodenko, Edina Rosta, Walter Kolch, Nicolae-Viorel Buchete

**Affiliations:** 1 School of Physics & Institute for Discovery, University College Dublin, Belfield, Dublin, Ireland; 2 Systems Biology Ireland, University College Dublin, Belfield, Dublin, Ireland; 3 School of Medicine, University College Dublin, Belfield, Dublin, Ireland; 4 Conway Institute, University College Dublin, Belfield, Dublin, Ireland; 5 Department of Chemistry, King’s College London, London, United Kingdom; Koç University, TURKEY

## Abstract

RASSF enzymes act as key apoptosis activators and tumor suppressors, being downregulated in many human cancers, although their exact regulatory roles remain unknown. A key downstream event in the RASSF pathway is the regulation of MST kinases, which are main effectors of RASSF-induced apoptosis. The regulation of MST1/2 includes both homo- and heterodimerization, mediated by helical SARAH domains, though the underlying molecular interaction mechanism is unclear. Here, we study the interactions between RASSF1A, RASSF5, and MST2 SARAH domains by using both atomistic molecular simulation techniques and experiments. We construct and study models of MST2 homodimers and MST2-RASSF SARAH heterodimers, and we identify the factors that control their high molecular stability. In addition, we also analyze both computationally and experimentally the interactions of MST2 SARAH domains with a series of synthetic peptides particularly designed to bind to it, and hope that our approach can be used to address some of the challenging problems in designing new anti-cancer drugs.

## Author Summary

We model the conformational changes and protein-protein interactions of enzymes involved in signaling along the Hippo pathway—a key molecular mechanism that controls the process of programmed cell death in eukaryotic cells, including cells affected by cancer. Combining modern computational modeling techniques with experimental information from X-ray crystallography and systems biology studies, can unveil detailed molecular interactions and lead to novel drugs. Here, we study the atomistic mechanisms and interactions between MST2 and RASSF-type kinases, through their respective SARAH domains—highly conserved, long, terminal α-helices, which play essential roles in the activation of MST kinases and, therefore, in modulating apoptosis. In spite of their key roles in mediating cell signaling pathways, there is little structural information available for the RASSF SARAH domains and their dimerization with the MST2 SARAH domains. In particular, the RASSF1A crystal structure is not available yet. Here, we model, refine and validate atomistic structural models of dimers of the RASSF1A and MST2 SARAH domains, studying the interaction and the dynamic behavior of these molecular complexes using homology modeling, docking and full atomistic molecular dynamics simulations. Experimentally, we validate our approach by designing a novel peptide that can disrupt effectively MST2 homo and hetero SARAH dimers.

## Introduction

There is an acute need for novel drug targets and strategies in the fight against cancer. New directions could emerge from exploring the tumor-suppressive RASSF signaling pathway and its downstream effectors, the MST1/2 kinases, which control tissue homeostasis by balancing cell proliferation and cell death through apoptosis [1–4]. The activation of MST1/2 kinase activity is regulated by either homo-dimerization or by interactions with scaffold proteins such as WW45 and different members of the RASSF family. The regulation of MST1/2 by RASSF scaffolds is a key event in this pathway, but remains poorly understood [3, 5]. The evidence we have so far indicates that the RASSF family members RASSF1A and RASSF5 (also known as NORE1 or RALP) are tumor suppressors that mediate apoptosis through different effectors including MST1/2 kinases, but their exact regulation by RASSF proteins is incompletely understood [6]. RASSF1A and RASSF5 regulate MST1/2 kinase activity by direct protein-protein interaction through their respective SARAH domains [7]. The SARAH domain is a long, conserved α-helix at the C-terminal end, known to be a key protein-protein interaction domain [8]. A comparative analysis of the RASSF family SARAH domains has been previously published by Chan et al. [9] and discussed also in Ref. [6]. We showed that other proteins that do not have a SARAH domain themselves, such as RAF1, could nevertheless also regulate MST1/2 kinase activity through direct binding to their SARAH domain [1, 10], confirming the importance of protein-protein interactions via the SARAH domain in the regulation of these kinases. In addition, RASSF proteins were shown to be able to activate or inhibit MST1/2 kinase activity upon heterodimerization [5].

**Fig 1:**
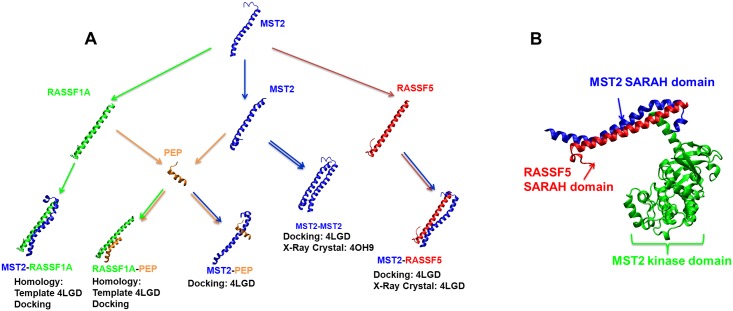
Dimeric interactions of SARAH domains. **(A)** Schematic representation of the principal monomeric and dimeric systems modeled in this study. Arrows represent the steps followed to construct our molecular models. **(B)** MST2-RASSF5 complex from crystal structure (PDB ID: 4LGD) showing the direct interaction between RASSF5 (red) and MST2 (blue) SARAH domains. The MST2 kinase domain (blue) is also resolved in the 4LGD crystal structure (the linker segment between the catalytic and SARAH domains is not resolved).

Given the importance that dimerization of MST1/2 and the RASSF proteins have on the regulation of MST1/2-dependent apoptosis, several studies have focused on the description of the interaction between RASSF5 and MST proteins through their SARAH domains, as summarized recently in Ref. [6]. Accordingly, crystal structures are available for the MST-RASSF5 SARAH domain dimers [11, 12]. The MST2-RASSF5 SARAH domain hetero-dimer (Fig 1) crystal structure was recently determined [11, 13], and further analysis of the MST2-RASSF5 interactions from the crystal structure was carried out from an experimental point of view [11]. However, only few studies considered the structure of the RASSF1A SARAH domain and its dimerization with the MST2 SARAH domain [14]. Importantly, the RASSF1A loss of expression is arguably one of the most frequent events in human solid tumors, and the characterization of RASSF1A-MST2 heterodimers could help to understand the important role of RASSF1A as a tumor suppressor [6].

In this study, we analyze the specific dimeric interactions between the helical SARAH domains of MST2 and RASSF enzymes. As indicated above, SARAH domains have been previously characterized in experimental *in vivo* studies of MST2, for example by using protein arrays to demonstrate their specific binding [1, 4]. However, this paper is our first modeling study that thoroughly examines the interactions between SARAH domains and SARAH—RASSFx interactions. Based on recently solved crystal structures of MST2-RASSF5 SARAH heterodimers, we use a combination of homology modeling, docking, molecular modeling and atomistic molecular dynamics (MD) methods, to construct and study a variety of models of MST2-MST2 homodimers, as well as of MST2-RASSF5 and MST2-RASSF1A SARAH heterodimers, and we identify the factors that control their high molecular stability. We also study the interaction of the MST2 SARAH domain with a *de novo* designed peptide, and demonstrate both via *in silico* modeling and experimentally that this peptide disrupts the MST2-RASSF1A interactions, as predicted. A summary, schematic representation of the principal monomeric and dimeric systems modeled in this study is illustrated in Fig 1A.

## Results

### Sequence analysis of RASSF SARAH domains

Fig 1B illustrates the MST2-RASSF5 complex from the only available crystal structure (PDB ID: 4LGD) [13] showing the direct interaction between the RASSF5 SARAH domain (red) and the MST2 SARAH domain (blue). The MST2 kinase domain (blue) is also resolved in the 4LGD crystal structure. To model the structure of RASSF1A SARAH domains, we performed multiple sequence analysis to infer similarities with structurally resolved homologues (e.g., RASSF5) from the same family. We performed a detailed sequence comparison of RASSF1-to-6 SARAH domains, using multiple sequence alignment obtained with the Clustal Omega software [15–17], which allowed us to infer its similarity with structurally resolved homologues (e.g., RASSF5) from the same family.

**Fig 2:**
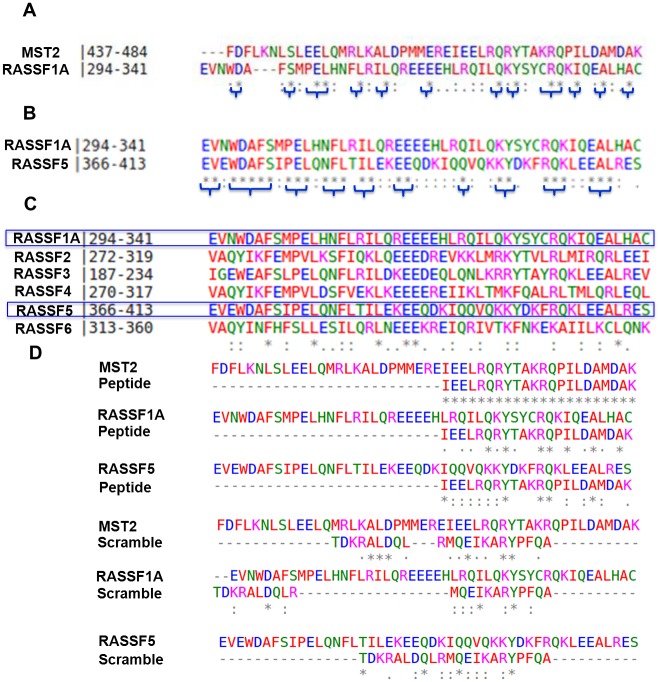
Sequence analysis. Sequence alignments obtained using Clustal Omega for SARAH domains of **(A)** MST2 and RASSF1A (31.4% sequence identity and 64.6% similarity), **(B)** RASSF1A and RASSF5 (54.1% sequence identity and 89.4% similarity), **(C)** RASSFn (with *n* ∈ [1,6]), and **(D)** MST2, RASSF1A, and RASSF5, as indicated, and the new peptide with its designed (marked “peptide”) and “scrambled” sequences. The colors correspond to: red = hydrophobic residue (small + hydrophobic (incl. aromatic—Y)); blue = acidic residue; green = hydroxyl + sulfhydryl + amine + G; magenta = basic residue—H. The asterisk (*) indicates a single, fully conserved residue. In the bottom row, the alignment results are represented as follows: a colon (:) indicates conservation between groups of strongly similar properties (i.e., scoring > 0.5 in the Gonnet PAM 250 matrix), and a period (.) indicates conservation between groups with weakly similar properties (i.e., scoring = < 0.5 in the Gonnet PAM 250 matrix).

Our analysis showed clearly that the RASSF SARAH domains contain multiple conserved sites (Fig 2A). Importantly, the pairwise alignment between RASSF1A and RASSF5 (Fig 2B) showed that their respective SARAH domains have 54.1% sequence identity and 89.4% sequence similarity. These values are significantly large (i.e., larger than in other recent studies using homology modeling in conjunction with MD simulations [18]), and this observation is both a strong motivation and a justification for using the crystal structure of the RASSF5 SARAH domain as a template for the homology modeling of the RASSF1A SARAH domain structure [6].

### MST2 SARAH domain as docking target

**Fig 3:**
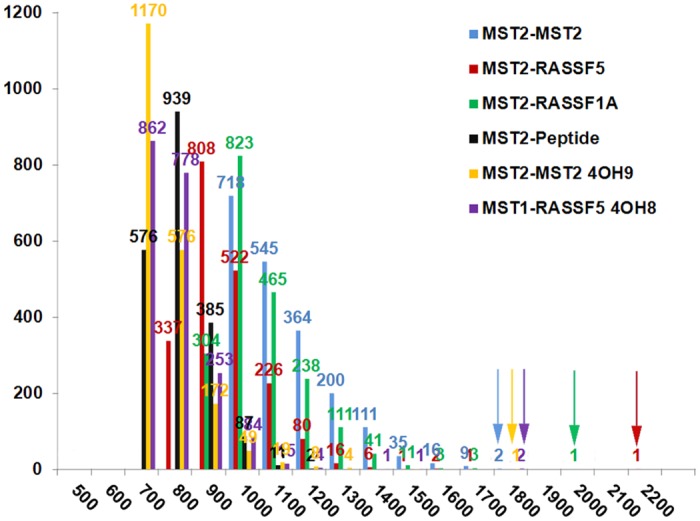
Molecular docking. Histogram of Zdock scores for MST2-MST2 (blue), MST2-RASSF5 (red), MST2-RASSF1A (green), and MST2-Peptide (black) dimers. Numbers indicate structures with similar scores. Arrows indicate Zdock scores obtained for crystal structures, when available, (i.e., red, yellow and purple), and also the highest scoring dimer structures used for subsequent MD refinement (i.e., blue & green, for cases where crystal structures are not available).

We continued our study by generating an atomistic model of the structure for the RASSF1A monomer built by homology modeling, using the RASSF5 crystal structure (4LGD: Chain G) as a template (Fig 1). Subsequently, the RASSF1A SARAH structure was docked onto the MST2 SARAH domain. A large number (approx. 2000) of possible dimer structures were generated using the Zdock program [19–21] (Fig 3). We note that coarse-grained modeling approaches, including docking, have been very successful in other recent computational approaches for studies of protein-protein interactions [22–25]. The Zdock scoring function values for MST2-MST2 (blue), MST2-RASSF5 (red), and MST2-RASSF1A (green) dimers were shown to be effective in identifying only a few most favorable binding modes, indicating that our strategy was valid for this study. Importantly, our analysis assigned high Zdock scores for cases when crystal structures are available (Fig 3 red, yellow and purple arrows), further validating our approach. Thus, we decided to use the docking-generated structures with the highest scoring function as initial models to study the MST2-RASSF1A dimer in the subsequent atomistic molecular dynamics study. However, we note that other recent docking approaches, including additional factors such as side chain flexibility, may further improve structural models before being refined with atomistic MD approaches [23, 24].

Interestingly, the MST2-RASSF1A structure with the highest scoring function corresponded to that in which both protomers are aligned in an anti-parallel topology, similar to those found in the crystal structure of MST2-RASSF5 [11, 13].

In addition to the analysis of the dimerization of different SARAH domains we also analyzed the structure of the interaction between the SARAH domain and a peptide that has been shown experimentally to bind to the MST2 SARAH domain [4]. In this case, similarly to MST2-RASSF1A, our docking study identifies only one structure standing out from the rest with the highest scoring function (Fig 3). Moreover, the top scoring 100 docking structures (out of a total set of 2000) appear to be clustered near the very same interface area, and no additional high-scoring contact area between MST2 and the designed peptide was found using Zdock.

### All-atom MD simulations of MST2 SARAH dimer structures

To validate and refine the results of our Zdock study we studied homo- and heterodimers of MST2-MST2, MST2-RASSF5 and MST2-RASSF1A using atomistic molecular dynamics (MD) with explicit water molecules.

A summary of the simulation types performed and analyzed here is given in Table 1.

**Table 1:** MD simulation parameters of the 12 atomistic systems of SARAH domain dimers (MST2-MST2, MST2-RASSF5, MST2-RASSF1A, and MST2-PEP, MST2-SCR, RASSF1A-PEP and RASSF1A-SCR) solvated using explicit TIP3P water molecules.

No.	Systems	Simulation time (ns)	No. of residues	Water molecules	Total no. of atoms	Initial dimensions (Å)
	**Dimers**					
1.	MST2-RASSF5	200	94	14,912	46,408	79x78x80
2.	MST2-RASSF1A	200	94	14,251	44,407	78x78x78
3.	MST2-MST2	200	94	22,543	69,298	90x90x90
4.	MST2-RASSF5 400K	160	94	14,912	46,408	79x78x80
5.	MST2-RASSF5 450K	70	94	14,912	46,408	79x78x80
6.	MST2-RASSF5 500K	55	94	14,912	46,408	79x78x80
7.	MST2-PEP_S_	200	64	13,205	40,729	75x75x76
8.	MST2-PEP_L_	200	70	12,775	39,620	75x75x75
9.	MST2-PEP_A_	200	70	14,684	45,357	78x78x78
10.	MST2-SCR	200	70	12,771	39,612	75x75x75
11.	RASSF1-PEP	200	69	13,914	43,029	77x77x77
12.	RASSF1-SCR	200	69	13,572	43,003	76x76x77

**Fig 4:**
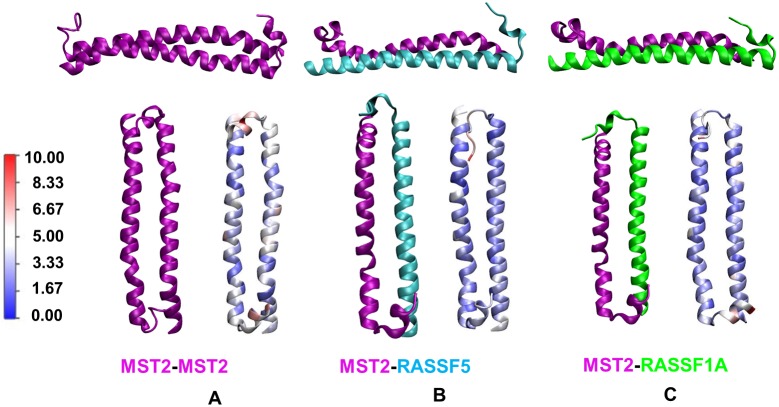
Structures of SARAH homo- and heterodimers. **Top**: Structures of **(A)** MST2-MST2 (purple-purple), **(B)** MST2-RASSF5 (purple-cyan) and **(C)** MST2-RASSF1A (purple-green) dimers. The MST2-RASSF5 dimer corresponds to the 4LGD crystal structure, and the other two correspond to our homology models. **Bottom**: Lateral views of the same structures from above. On the r.h.s., each structure was colored according to its residue-level RMSF value calculated using MD trajectories.

Fig 4 shows the three dimers in our MD study: MST2-RASSF5 from crystal structure (4LGD), MST2-MST2, and MST2-RASSF1A. The structure of the MST2-MST2 homodimer modeled corresponds to a parallel alignment of both protomers (Fig 4A). The crystal structure of the MST2-RASSF5 dimer was refined with MD (Fig 4B). The structure of the MST2-RASSF1A dimer selected was the one with the highest scoring function, which additionally presented a parallel alignment of both SARAH domains (Fig 4C).

Cα root-mean-square deviation (RMSD) values for all four atomistic systems, both homo- and hetero-dimers, are shown in Fig S1 in S1 File. We observed that the RMSD values in both systems are converged and remain stable along our entire trajectories. Additionally, solvent accessible surface area (SASA) was estimated in order to assess the solvation properties of hetero-dimers. Fig S1 in S1 File shows that in all cases, SASA values also converged, remaining almost constant and not showing any significant changes along the trajectories. Thus, the buried surface at the interface between protomers upon dimer formation is almost constant along the trajectory.

Root-mean-square fluctuation (RMSF) values for all four dimer types were calculated in order to gain insight in the flexibility of the SARAH domains upon complex formation (Fig 4, lower panel r.h.s.). In general, all the residues fluctuate very little with respect to the initial structure (RMSF depicted as blue), with the exception of the C-terminal loop (white-red) which presents more flexibility. In the case of the MST2-MST2 dimer, the residues located in the centers of the helices presented more mobility as compared with the corresponding residues in the RASSF1-6 dimers.

We carried out additional MD simulation at different temperatures over the MST2-RASSF5 crystal structure dimer, at 400 K, 450 K and 500 K. Those trajectories showed that even at 400 K, MST2-RASSF5 dimer was stable at least during 160 ns (see Fig S2 in S1 File), while in the 450 K and 500 K, both SARAH domains became unstable after 30–50 ns.

We also calculated the interaction energy between protomers within the dimers for all the systems studied. The total interaction energy (see Fig S3A in S1 File, black line) was defined to include electrostatic (green) and van der Waals (vdW, blue) interactions. This analysis showed that the dominant term is electrostatic (Fig S3A in S1 File). The vdW term remains constant along the trajectory in all the cases, and it has a relatively minor contribution. The electrostatic interaction term accounts for -527.5 ± 69.6, -483.4 ± 77.1, -426.9 ± 71.5 kcal/mol for MST2-MST2, MST2-RASSF5, and MST2-RASSF1A dimers respectively, while the vdW term accounts for only -149.1 ± 7.1, 118.4 ± 7.6, 114.6 ± 7.2 kcal/mol.

These results based on MD simulations also show that the total interaction energy of the MST2-MST2 dimer is only marginally more stable than the MST2-RASSF5 and MST2-RASSF1A dimers (Fig S3 in S1 File). The average total interaction energy for MST2-MST2 is -676.5 ± 67.8 kcal/mol, while in the case of MST2-RASSF5 and MST2-RASSF1A, it accounts for -601.7 ± 75.0 and -541.5 ± 70.4 kcal/mol, respectively. Additionally, the total interaction energy distributions, calculated for each dimer along its trajectory (Fig S3B in S1 File), show that all three dimers present similar populations within the same range of energies.

To further probe the interaction preferences between the SARAH domains of MST2 and RASF1A and RASSF5, we have also used six different contact potentials to estimate the normalized contact energies based on structures obtained from MD trajectories in each case. While contact potentials offer only rough approximations for the relative stability of molecular structures, have been remarkably successful in other studies of protein-protein interactions [22, 24]. In Fig S11 in S1 File we illustrate a comparison of histograms of residue-residue contact potential values from 20 x 20 contact potential matrices [26] developed by Hinds and Levitt (HL [27]), Betancourt and Thirumalai (BT [28]), Miyazawa and Jernigan (MJ-99 [29]), Skolnick et al. (SJKG [30], and SKO from Ref. [31]), and Tobi et al.[32] (TSLE from Ref. [32]). These normalized contact potentials are further used in Fig S12 in S1 File to calculate relative contact potentials values for structures of MST2-RASSF1 (notation MR1 on the horizontal axis), MST2-MST2 (MM) and MST2-RASSF5 (MR5) dimers. The calculations were performed for the six popular contact potentials represented Fig S11 in S1 File for three cases: (A) dimer structures before MD, (B) the dimer structures from our MD simulations corresponding to frames with the smallest RMSD values compared to the average over the respective trajectory (RMSD_ave_), and (C) the dimer structures from the same MD trajectories but corresponding to frames with the largest RMSD_ave_ (to illustrate that even in this case the relative values for MR1 are still smaller than for MM and MR5 dimers). In Fig S12 in S1 File, a residue-residue contact cut-off distance of 5.5 Å between side-chain atoms was used, though we obtained similar results when using cut-off distances of 5.0 Å and 6.0 Å on the same structures.

Interestingly, as shown in Fig S12 in S1 File, there is a remarkable agreement between results obtained for the six different contact potentials used here, in spite of their diversity and well-known approximate accuracy due to their intrinsic coarse-grained character. Nevertheless, in agreement with the atomistic MD energies, the results suggest that all three dimer types have similar stabilities, though this time MST2-RASSF1A dimers appear to be marginally more stable. This is in agreement with experimental observations that both RASSF1A and RASSF5 SARAH domains could disturb competitively MST2 SARAH homodimers.

As an additional probe of the relative stability of the various dimeric systems for which we have MD simulations available, we have also calculated potential of mean force (PMF) profiles as presented in Figs S14 and S15 in S1 File. In Fig S14 in S1 File, results are presented for the MST2-MST2, MST2-RASSF1 and MST2-RASSF5 systems calculated from the corresponding dimer all-atom MD simulations using the recent dynamic histogram analysis method (DHAM) method [33]. The profiles were calculated for the distance between carbon alpha (CA) atoms of the two monomers that has the smallest average value along the corresponding MD trajectory. To probe convergence, in Fig S14 in S1 File the PMF profiles are presented for (A) the full trajectory, (B) the first third of the data, (C) the second third, and (D) the final third of the data. The first 20 ns (i.e., ~10%) of data from each trajectory were not included in this analysis. Though free energy calculations for interactions between large molecular complexes are notoriously difficult, the calculated PMF profiles suggest that the RASSF1 SARAH domains could bind better to monomeric MST2 SARAH domains than MST2 itself (e.g., the MST2-RASSF1 PMF values in Fig S14 in S1 File, red curves, appear to have narrower profiles when compared to MST2-MST2, blue curves, though this effect is weaker in the MST2-RASSF5 case, yellow curves).

Similarly, PMF profiles for the MST2-PEP_A_, MST2-PEP_L_, MST2-PEPs, MST2-SCR, RASSF1-PEP, and RASSF1-SCR systems calculated from the corresponding dimer all-atom MD simulations using the DHAM method are also presented in Fig S15 in S1 File [33]. Here, the PMF profiles illustrate clearly the trends discussed in detail above (e.g., most notably that scrambled peptides have a lower dissociation barrier than their corresponding counterparts).

In order to provide further information on the MST2-MST2 homodimer, we have carried out a docking study (see yellow plot in the histogram in Fig 3) of the MST2-MST2 dimer using the crystal structure available (4HO9). We have evaluated the structural differences between the 4HO9 structure and the results from docking using 4HO9 as template. Our results (see in Fig S13 in S1 File upper panel) indicate that the differences between both docked and crystal structures are remarkably small, corresponding to alpha carbon (CA) RMSD of 2.208Å. Furthermore, we have repeated the experiment, comparing the dimer obtained from docking using 4LGD (MST2 monomer) and the available 4HO9 crystal structure (see in Fig S13 in S1 File lower panel). Once more, the docking results are in very high agreement with the crystal structure, with a CA RMSD of 2.096 Å.

**Fig 5:**
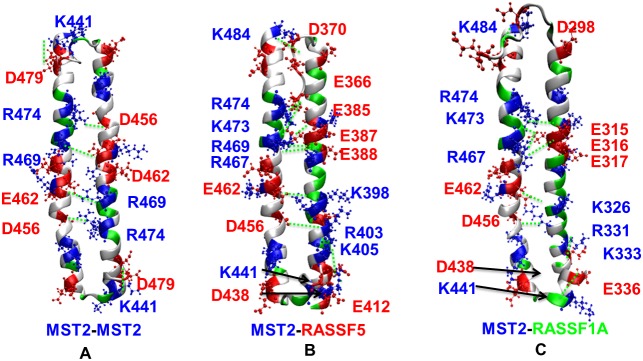
Charged residues and salt bridges in SARAH dimers. Representation of charged residues and salt bridges present within the three SARAH dimer types: (A) MST2-MST2, (B) MST2-RASSF5, and (C) MST2-RASSF1A. Green dashed lines correspond to salt bridges.

In order to gain additional insight of the different interactions that occur upon complexation between the protomers, salt bridges and hydrogen bonds were analyzed. A deeper analysis of the MD structures revealed several salt bridges present in these dimers (see Fig 5 and Table 2).

**Table 2:** Salt bridge dynamics in SARAH domain dimers (MST2-MST2, MST2-RASSF5 and MST2-RASSF1A). Salt bridge contacts formed in more than 10% of the trajectory are highlighted as bold.

MST2	MST2	RASSF5	RASSF1A
**K 441**	D479 (0.55%)	**E412 (41.7%)**	E366 (0.1%)
**R 451**			
**R 467**		E385 (1.3%)	**E316 (91.1%)**
		**E388 (87.7%)**	E317 (0.6%)
**R 469**	**E462 (74.5%)**	E366 (0.5%)	
**K 473**		**E366 (15.4%)**	E315 (1.3%)
		E387 (1.7%)	
**R 474**	**D 456 (90.6%)**	**E385 (28.9%)**	**E316 (81.9%)**
		**E388 (80.0%)**	
**K 484**		D370 <0.1%	D298 (0.3%)
**D 438**		K405 (1.8%)	K333 (3.5%)
**D 456**	**R474 (90.6%)**	R403 (0.5%)	**R331 (98.1%)**
**E 462**	**R469 (74.5%)**	**K398 (75.1%)**	**K326 (60.9%)**
**E 465**			
**D 479**	K441 (0.55%)		

The MST2-MST2 dimer presents symmetrical interactions between K441-D479, D456-R474 and E462-R469, with the last two pairs being dominant in terms of shorter distance along the trajectory (see Fig S4 in S1 File). This is because the first salt bridge (K441-D479) belongs to the terminal ends of both helixes and it is very flexible, making this salt bridge less stable. The MST2-RASSF5 dimer contains a large number of salt bridges, and a total of 12 interactions were found, while in MST2-RASSF1A dimer 9 salt bridges were located. In the MST2-MST2 dimer the electrostatic interactions are mainly between the two pairs R469-E463 (*P*_*c*_ = 0.745) and R474-D465 (*P*_*c*_ = 0.906), where *P*_*c*_ is the probability of formation of the corresponding contact as estimated from our MD trajectories. In the case of MST2-RASSF5, more salt bridges come into play accounting for the highest contact propensity along the MD simulation: R467-E388 (*P*_*c*_ = 0.877), K473-E366 (*P*_*c*_ = 0.154), R474-E385 (*P*_*c*_ = 0.289) which are competing with the formation of R474-E388 (*P*_*c*_ = 0.800) and E463-K398 (*P*_*c*_ = 0.751). Furthermore, the MST2-RASSF1A dimer showed similar interactions with the MST2-RASSF5 dimer, R467-E316 (*P*_*c*_ = 0.911) and R474-E316 (*P*_*c*_ = 0.819), but also some similarities with the MST2 homodimer, D465-R331 (*P*_*c*_ = 0.981) and E462-K326 (*P*_*c*_ = 0.609). In view of these results, we concluded that R474 (MST2) and E462 play an important role in all these dimers, based on the *Pc* values observed for those amino acids. R467 and D456 are also highlighted as main “anchoring” contacts between dimers, especially between RASSF1-6 and MST2.

Our structural analysis identified the charged residues and salt bridges involved in the formation of the three models (Fig 5): (A) MST2-MST2 (blue-blue), (B) MST2-RASSF5 (blue-red), (C) MST2-RASSF1A (blue-green). As observed, electrostatic interactions play a primary role in controlling the assembly and stability of MST2 homo- and heterodimers. Notably, in MST2-RASSF5 and MST2-RASSF1A interactions the two antiparallel helices present a significant charge complementarity between their N-terminal and C-terminal regions. The time-dependent dynamics of several representative salt bridges is illustrated in the Fig S4 in S1 File.

**Fig 6:**
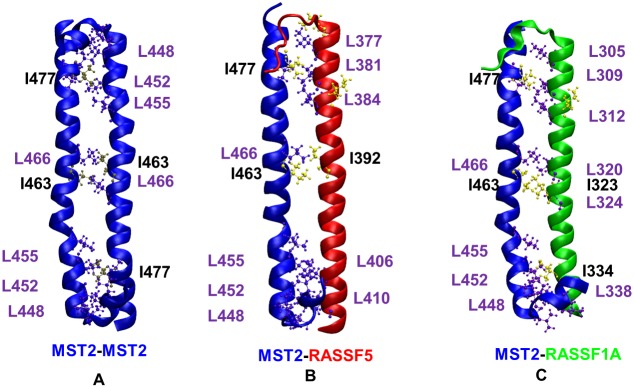
Hydrophobic interactions in SARAH dimers. Representation of Ile (yellow)–Leu (purple) contacts present within the three dimer types **(A)** MST2-MST2, **(B)** MST2-RASSF5 and **(C)** MST2-RASSF1A.

Recent studies have pointed out that hydrophobic isoleucine-leucine (ILE-LEU) pair interactions mediating packing between α-helixes also can contribute to the stabilization of such dimers [34]. Therefore, we analyzed the ILE-LEU pairs within the dimers, and several were found in MST2-MST2 and MST2-RASSF dimers (Fig 6). The number of those pairs and the particularly strategic location at the N-terminal, C-terminal and in the center of the dimer suggest a large stabilization. Thus, we conclude that these ILE-LEU pair interactions together with the salt bridges are the main elements responsible for dimer stability and, possibly, formation in the MST2-RASSF1-6 and MST2-MST2 SARAH domains.

In addition to the salt bridges discussed above, several hydrogen bonds (HBs) were identified between protomers, which also contribute to the stability of dimers. The total number of hydrogen bonds (*N*_*HB*_, calculated using VMD [35]), that occur along the MD simulation within the dimers, is 16, 31 and 24 for MST2-MST2, MST2-RASSF5 and MST2-RASSF1A. Table 3 shows the total occupancy of those HBs along the whole MD trajectory, including the donor and acceptor motif. Furthermore, the time-dependent dynamics of the hydrogen bonds is depicted also in Fig S5 in S1 File. We observed that MST2 key hydrogen donors correspond to Y470 and R474 amino acids that are involved in HB within all the dimers studied here. Other main important contributions arise from the residue R467, which presents hydrogen bonds with Q389 (12.5%) and E388 (52.0%) in RASSF5, and with E316 (48.3%) of RASSF1A, and from the residue R469 that presents hydrogen bonds with E462 (98.7%) in MST2-MST2 dimer.

**Table 3:** Hydrogen bond (HB) dynamics in SARAH domain dimers (MST2-MST2, MST2-RASSF5 and MST2-RASSF1A). Only HBs with more than 1% occupancy are shown. HBs formed in more than 10% of the trajectory are highlighted in bold. *Top*: MST2 domains acting as an HB donor, *Bottom*: MST2 acting as a HB acceptor.

MST2 (HB Donor)	MST2	RASSF5	RASSF1A
**K441**		**E412 (21.2%)**	
		E408 (4.0%)	
**E446**			
**Q449**			
**R467**		**Q389 (12.5%)**	**E316 (48.3%)**
		**E388 (52.0%)**	
**R469**	**E462 (98.7%)**	E366 (3.0%)	E294 (4.0%)
**Y470**	**E462 (59.6%)**	**E387(24.2%)**	**H319 (23.6%)**
			E315 (9.2%)
**K473**		E366 (7.0%)	E294 (1.1%)
**R474**	**D456 (47.4%)**	**E388 (45.4%)**	**E316 (54.8%)**
		E385 (9.8%)	**Q313 (11.1%)**
**K484**	L443 (6.0%)	F372 (3.0%)	**D298 (18.9%)**
	K441 (4.8%)	D370 (9.4%)	F300 (3.3%)
MST2 (HB Acceptor)			
**K441**	K484 (5.2%)	S413 (1.3%)	
**L443**	K484 (4.5%)	**S413 (17.1%)**	
**D438**			K333 (1.2%)
**Q449**			
**D456**	**R474 (25.4%)**	R403 (1.6%)	**R331 (62.1%)**
**E462**	**R469 (88.6%)**	**Y399 (58.7)**	**K326 (28.8%)**
	**Y470 (56.0%)**	**K398 (38.6)**	**Y327 (51.2%)**
**E465**			
**Y470**		K391 (1.8%)	
**P476**		W369 (2.3%)	

Additionally, we calculated the hydrophobic (SASA_H_) and hydrophilic (SASA_P_) fractions of the solvent accessible surface area (SASA) for the representative structure of each system in order to gain insight on the hydrophobic interactions (see Fig S6 in S1 File). From this calculation, it was not clear whether hydrophilic (pink) or hydrophobic (green) exposed areas are predominant. To clarify this point we quantified the total exposed area in these dimers, together with the SASA_P_ and SASA_H_ values of the representative structures using the GetArea program [36]. These values, given in Table 4, show that the MST2-MST2 dimer has the largest exposed hydrophobic surface, while MST2-RASSF5 presents the smallest one.

**Table 4:** Hydrophobic (SASA_H_) and hydrophilic (SASA_H_) solvent accessible surface area (SASA), in Å^2^, for each of the three types of dimers.

	SASA_H_	SASA_P_	*Total SASA*	*% Hydrophobicity*
MST2-MST2	4391.6	2864.7	7256.3	60.5%
MST2-RASSF5	4170.8	2990.4	7161.3	58.2%
MST2-RASSF1A	4426.2	2940.1	7366.2	60.1%

### Disruptor peptide interacting with MST2 and RASSF1A SARAH domains

In previous work, the interaction between RAF1 and MST2 protein was studied from experimental and computational point of view showing that MST2 coordinates crosstalk between the mitogenic Raf and pro-apoptotic MST2 pathway [4]. This study showed that a 17-mer peptide designed based on the binding site of RAF1 to the MST2 SARAH domain was able to disrupt RAF1-MST2 dimerization. Understanding how such peptides bind and disrupt the dimerization process is key for future development of anti-cancer drugs that can activate MST2 by releasing it from the inhibitory interaction with RAF1. In addition, it will help to design peptides that either improve or do not affect RASSF1A or MST2 interactions, as desired, or could also simultaneously disrupt RASSF1 and MST2 dimerization. For that reason, we carried out a computational study of the possible interactions between the so-called disruptor peptide and the MST2 and RASSF1A SARAH domains. In order to perform this study, we used homology modeling and docking studies to obtain the initial structures and, in a subsequent step, full atomistic MD simulations to validate and analyze these interactions.

Four different systems were tested. We first used the disruptor peptide (PEP_S_) with the sequence “**RYTAKRQPILDAMDAK**” corresponding to the minimal sequence of MST2 known to interact with RAF1 [1]. A longer peptide (PEP_L_) “**IEELRQRYTAKRQPILDAMDAK**”, including flanking sequences of MST2-RAF1 interaction domain, was also tested in order to see the influence of the length of the peptide on the interaction. Both initial dimeric structures (MST2-PEP_S_ and MST2-PEP_L_) were obtained from the highest scoring structure in the docking study. In addition, PEP_L_ was aligned to the original MST2-MST2 SARAH domains (PEP_A_). Finally, we also studied a control peptide, where the PEP_L_ sequence was scrambled, “**TDKRALDQLRMQEIKARYPFQA**”. As the RAF1 binding domain overlaps with the RASSF1A binding in the MST2 SARAH domain we also investigated the structures of RASSF1A-PEP_A_ and RASSF1A-SCR dimers (see Fig S9 in S1 File for sample structures of RASSF1A-SCR dimers along the 200 ns MD trajectory).

Fig S7 in S1 File shows the RMSD of the four MST2-Peptide systems studied. When we compared the interactions between PEP_S_ and PEP_L_ and the MST2 SARAH domain we observed that in the latter, the dimer was more stable than in the former. In fact, the fluctuation of PEP_S_ around the MST2 SARAH domain is larger than PEP_L_. Once the peptide is aligned (PEP_A_) with the MST2 SARAH domain in the MST2-MST2 SARAH domain dimer, the peptide remains almost constant and no significant deviation from the initial structure can be found after 200 ns. This was shown by the stable RMSD along the MD trajectory.

Next, we compared MST2-PEP_L_ with the MST2-SCR system, both from the best scoring docking structures, to gain insight in the structural stability of both systems. We observed that the RMSDs with respect to the initial structure (black) converge to the same values, between 5–7 Å, while with respect to the average structure (red) MST2-PEP_L_ converges to a more stable structure (2–3 Å), and MST2-SCR seems to drift towards RMSD of 4 Å. This may be indicative of the decreased stability of the SCR peptide versus the PEP_L_. Furthermore, the highly structural stability of MST2-PEP_A_ corroborates the experimental observation that the PEP but not the SCR can disrupt RAF1-MST2 interaction by blocking the RAF1 interaction domain of the MST2 SARAH domain.

Our simulations showed that the interaction between RASSF1A and PEP_A_ dimer, the structure remains almost constant along the MD trajectory with a slight variation at 170 ns due to the fluctuation of the C-terminal part of the RASSF1A SARAH domain.

For RASSF1A-SCR dimer the picture was quite different, and RMSD_ave_ in respect to the average structure keeps fluctuating (see Fig S7 in S1 File). This is due to the unfolding of the scrambled peptide along the trajectory, which destabilizes this dimer (see Fig S8 in S1 File). This is also revealed by the large SASA variation for the RASSF1A-SCR dimer, indicating that PEP which was designed using the MST2 SARAH domain sequence could also bind to the homolog sequence of RASSF1A SARAH domain, since both SARAH domain sequences have 31.4% identity and 64.6% similarity (Fig 2A).

**Fig 7:**
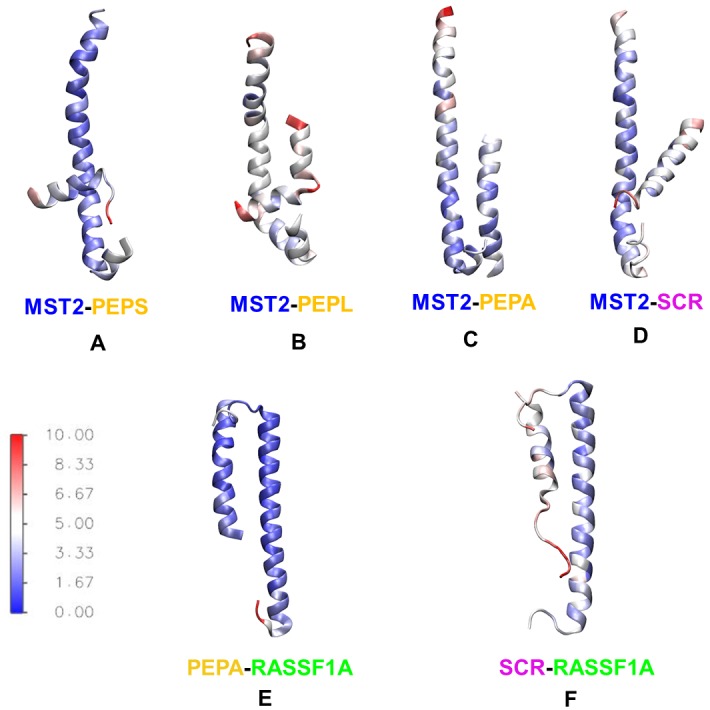
Interactions of MST2 SARAH domains with designed peptides. **Top**: Structures of **(A)** MST2-PEP_S_, **(B)** MST2-PEP_L_ (blue-red), **(C)** MST2-PEP_A_ and MST2-SCR dimers. **Bottom**: Structures of **(E)** RASSF1A-PEP_A_, and **(F)** RASSF1A-SCR dimers. The color scale represents residue-level root mean square fluctuations (RMSF) calculated for each dimer from MD data, with blue and red indicating rigid and flexible amino acids, respectively.

Root mean square fluctuation (RMSF) values for all six dimer types were calculated in order to gain insight into the flexibility of the SARAH domains upon complex formation (Fig 7). The differences between aligned structures for PEP_A_ and the scramble SCR one are notable. While the aligned peptide, PEP_A_, is quite rigid along the trajectory, the SCR peptide shows more flexibility and visits less stable conformations.

**Fig 8:**
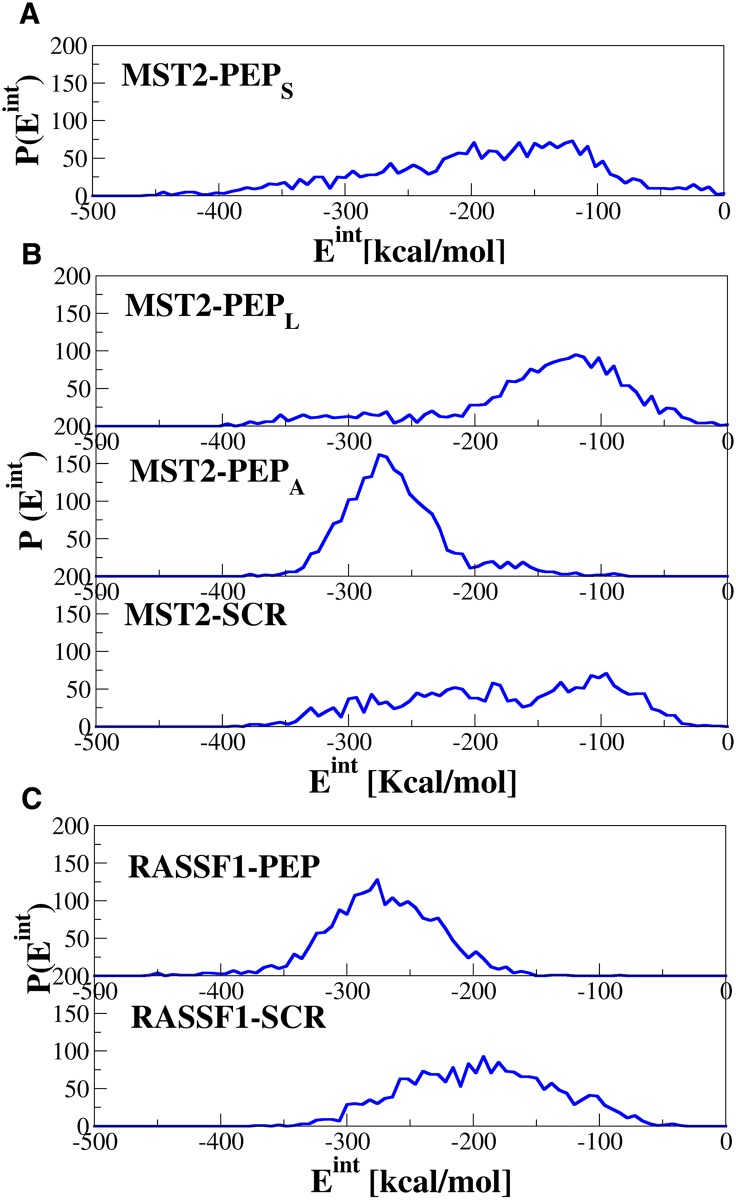
Interaction energies of dimers including MST2 and RASSF1A SARAH domains and designed peptides. Distributions of the total interaction energy between the two protomers for MST2-PEP_S_, MST2-PEP_L_, MST2-PEP_A_, MST2-SCR, RASSF1A-PEP_A_ and RASSF1-SCR dimers.

In order to provide further insight in the relative stability of dimers containing PEP and SCR peptides, we analyzed the interaction energy along their corresponding MD trajectories (Fig 8 and Fig S10 in S1 File). Only the dimeric systems (MST2-PEP_L_, MST2-PEP_A_, and MST2-SCR) and (RASSF1A-PEP_A_, and RASSF1-SCR) have the same overall sequence composition and thus can be compared exactly to each other, though the estimated energy corrections for different residue composition are very small in this case for all systems.

In Fig S10 in S1 File is shown the total interaction energy (black) for the systems considered. Clearly, the MST2-PEP_A_ system presents the strongest interaction energy. Again, once the peptide is aligned with the MST2 SARAH domain, the structure is very stable. In the case of MST2-SCR, the interaction energy drifts and decreases along the trajectory, showing a destabilization of the dimer. Interestingly, a similar behavior is found in the RASSF1A-PEP complex, in which this dimer is very stable. This further indicates that the peptide can also interact favorably with the RASSF1A SARAH domain and hence can disrupt the MST2-RASSF1A dimerization. Furthermore, the interaction energy distribution along the trajectory (see Fig S10 in S1 File) clearly shows that the MST2-SCR system presents a broad distribution profile with only a small population at more negative energies. However, in the MST2-PEP_A_ distribution a peak of the energy values between -350 and -250 kcal/mol suggests a higher stability of the MST2-PEP_A_ system as compared to MST2-SCR. Similar relative interaction strengths have been found for the RASSF1A-PEP and RASSF1A-SCR systems confirming the higher relative stability of dimers containing PEP peptides.

**Fig 9:**
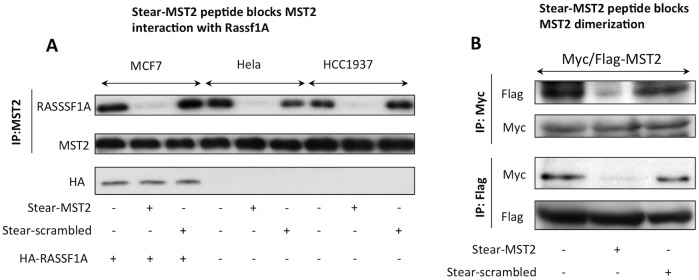
Stear-MST2 disruptor peptide blocks MST2 interactions with (A) RASSF1A, and (B) with other MST2. **(A)** MCF7 cells were co-transfected with Myc-tagged and Flag-tagged MST2. 48 h after transfection, cells were incubated with 10 μM of N-terminal stearoylated peptides (stear-MST2 or stear-scrambled as control) for 1 h. The cells were lysed, and MST2 was immunoprecipitated (IP) with antibody against the Flag-tag and Western blotted (WB) with anti-Myc tag antibody in order to detect MST2 homodimers. The experiment was also repeated in a second way using an anti-Myc tag antibody for IP and anti-Flag for Western blotting. **(B)** The cells lines indicated here were incubated with 10 μM of N-terminal stearoylated peptides (stear-MST2 or stear-scrambled as control) for 1 h. The cells were lysed, and endogenous MST2 was IP and WB for associated RASSF1A. As MCF7 cells do not express endogenous RASSF1A, they were transfected with an HA-tagged RASSF1A expression vector.

Since the computational simulations suggest the existence of stable dimers between MST2 and the disruptor peptide, we decided to test it experimentally in order to validate our atomistic models and prove the appropriateness of this approach for *in silico* testing of small molecules that target SARAH domain binding. The disruptor peptide was designed to include the minimal binding interface between RAF1 and MST2 as mapped by peptides arrays, and we showed that it disrupts this interaction very effectively [4]. In order to test our prediction that this peptide could also disrupt the RASSF1A-MST2 dimer, we performed co-immunoprecipitation experiments in MCF7 cells treated with the PEP or the SCR. Our experiments clearly demonstrated that this peptide disrupts the MST2-RASSF1A dimerization (Fig 9A). Moreover, when we tested the effects of this peptide on the MST2-MST2 interaction we also saw a disruption of the MST2 homodimerization (Fig 9B) that could have important effects in the activation of the kinase activity of this protein. All together, these experiments confirm the accuracy of our atomistic models and validate the molecular modeling and simulation methods used in this work.

## Discussion

SARAH domains are highly conserved throughout evolution and mediate the protein-protein interaction between the MST1/2 kinases and members of the RASSF family. RASSF1A and RASSF5 are key *bona fide* tumor suppressor genes, whose protein products have been shown to regulate MST1/2 kinase activity [4, 37]. The effect of RASSF1A and RASSF5 over MST1/2 activation is mediated by heterodimerization through the SARAH domain of these proteins. Thus, for a complete description of how RASSF1A and RASSF5 regulate MST2 we need to understand first how the SARAH domains mediate the formation of these complexes [6]. Previous studies have focused mainly on the RASSF5-MST2 dimer using crystal structures, NMR spectroscopy and performing limited computational analysis based only on structure minimization rather than using extensive atomistic MD. This approach has significant limitations since it does not allow the study of the dynamical behavior of protein dimers under realistic conditions including explicit solvent molecules. In order to get a better understanding of the structure of the SARAH dimers we used a combination of homology modeling, docking, molecular modeling and atomistic molecular dynamics (MD) methods, to construct and study a variety of models of MST2 homodimers, and of MST2-RASSF SARAH heterodimers, including the important case of RASSF1A-MST2 dimers. In the first step, based on the high sequence identity and similarity between RASSF1A and RASSF5, we used the MST2-RASSF5 dimer structure (Fig 1) based on the available crystal structure (PDB ID: 4LGD) [13] as a template for building new structural models for the RASSF1A SARAH domain interacting with MST2, as well as for MST2 homodimers. In the second step, to validate our models and also to search for alternative solutions, we used molecular docking to generate a broad variety of dimeric homo and heterodimer structures. Subsequently, we used atomistic MD simulations including explicit water representation to test the stability of our dimer models that have been previously ranked as best in the docking stage. With this approach, we showed that our models present a significant stability when probed with atomistic MD simulations, justifying additional experimental tests.

Importantly, in addition to the study of SARAH dimers we also simulated the structure of a synthetic peptide that was purposely designed to be a strong MST2 binding partner, and was used in our previous experiments. This peptide was designed to disrupt the RAF1-MST2 dimer, and to potentially activate MST2 kinase activity by binding to a short sequence of the MST2 SARAH domain where RAF1 interacts with this protein. Interestingly, our new simulations presented here show a different scenario for the effect of this peptide on the formation of RASSF1A-MST2 and MST2-MST2 dimers. Essentially, our new simulations indicate that the peptide would also prevent the formation of the RASSF1A-MST2 dimers, in addition to inhibiting the formation of MST2 homodimers. Importantly, we have validated experimentally these predictions of our *in silico* results, indicating strongly that we have correctly characterized the structure of the SARAH domain dimers, and confirming the relevance of our approach to the study of the structure of SARAH domains in particular, and of other protein-protein interactions in general. The molecular interaction mechanisms revealed here also shed new light on how RASSF1A regulates the MST2 kinase activity via dimer formation.

Significantly, our specific findings regarding SARAH domain interactions, together with the general methods used in this work, can help to design more effective strategies to target human cancer tumors (e.g., by deregulation of their RASSF1A/MST2 signaling networks). We also hope that this study is a first step towards integrating atomistic-level mechanistic information about the structures and conformational dynamics of proteins interacting through SARAH domains, with information available on their system-level functions in cellular signaling.

## Methods

### Experimental methods

#### Cell lines and transfections

MCF7, HeLa and HCC1937 where grown in Dulbecco's Modified Eagle's medium (DMEM) supplemented with 10% foetal calf serum (Gibco-BRL) and 2mM L-glutamine. MCF7 cells were transfected using Lipofectamine following the manufacturer’s instructions.

#### Immunoprecipitation and immunoblotting

Cells were lysed as previously described [1] 48 hours after transfection. Lysates were incubated with the different antibodies for 4h at 4°C. Immunoprecipitates were washed and resolved by SDS-PAGE as previously described [17].

#### Disruptor peptides

The peptides used in this study for affecting the dimer stability (see Fig 2D) were synthesized by the Cancer Research UK peptide Synthesis Laboratory and have been characterized recently [4].

### Homology modeling and docking

The initial structure of an MST2-RASSF5 SARAH domain dimer was constructed based on the crystal structure PDB ID: 4LGD, chain C and G for MST2 and RASSF5, respectively [13].

To our knowledge, there is no crystal structure of RASSF1A. Therefore we have used the sequence that is available from SwissProt [38] (Uniprot Q9NS23). Searches for homologous protein were carried out using UniProt [39] and the RCSB PDB data banks. The Clustal Omega program [15–17] was used for sequence alignment. A homology model of the RASSF1A SARAH domain was built using the comparative modeling environment, SWISS-MODEL [40–43]. The PDB structure 4LGD (chain G) for the RASSF5 SARAH domain was used as a template. In order to get an optimal packing structure between RASSF1A and MST2 SARAH domains, a docking study was carried out, which used the most probable structures (i.e, with the highest scoring function) generated by the Zdock server [19–21].

### All-atom validation of docking structures and refinement using MD

Four systems were prepared for atomistic MD simulations: one based on the crystal structure for the MST2-RASSF5 SARAH domain dimer, one with homology model structures for the MST2-RASSF1A SARAH domain dimer, one corresponding to the homodimer MST2-MST2 using the MST2 protomer structure from crystal structure and docking another copy of the MST2 protomer, and finally one with a homology model for the designed disrupting peptide.

Each system was solvated with explicit TIP3P water molecules [44] prior to minimization, heating and equilibration. The total number of atoms for each system including water molecules is reported in Table 1.

MD simulations were performed using the NAMD software [45] with the CHARMM36 force field [46]. All the atomistic MD simulations were performed in the NPT ensemble (i.e. constant number of atoms, pressure and temperature), using periodic boundary conditions, as in our similar recent MD studies [47–49]. We used the modified Nosé-Hoover Langevin piston method implemented in NAMD [50, 51] with damping time of 0.1 ps, while maintaining a pressure of 1.01325 bar. The temperature was set to 310 K and controlled using a Langevin thermostat with a 1 ps^-1^ damping coefficient. Ions were added using the automatic script provided in VMD [35] to achieve a neutral pH. The Particle Mesh Ewald method was used to include electrostatic effects [52]. The switching distance for non-bonded electrostatic and van der Waals interactions was 9.5 Å with a cut-off distance of 12 Å. The integration time step was 1 fs.

A summary of the simulations performed and analyzed here is given in Table 1. To address convergence, errors were estimated by block averaging and all the MD simulations were performed at least twice longer than needed to obtain the average values reported in each case. In addition, the PMF profiles presented in Figs S14 and S15 in S1 File were calculated and presented for different trajectory segments (e.g., the first, second and last third of each trajectory shown in Fig S14B, S14C, and S14D in S1 File, respectively, and the first and second half of each corresponding trajectory of Fig S15B and S15C in S1 File), as well as for the entire trajectory data (see Fig S14A and S14B in S1 File).

## Supporting Information

S1 File Supplemental data showing Figures S1 to S15 and the corresponding legends and references. (PDF)
