# Supplementary material for: SARAH Domain-Mediated MST2-RASSF Dimeric Interactions
Source: PLoS Comput Biol. 2016 Oct 7;12(10):e1005051. doi: 10.1371/journal.pcbi.1005051 (PMC5055338; doi:10.1371/journal.pcbi.1005051)
Supplement: S1 File — (PDF) [file pcbi.1005051.s001.pdf]

Supporting material (S1 File) for:

SARAH Domain-mediated MST2-RASSF Dimeric Interactions

Goar Sánchez-Sanz[1,Ω], Bartłomiej Tywoniuk[1,Ω], David Matallanas[2,4], David Romano[2], Lan K. Nguyen [2,Ψ], Boris N. Kholodenko[2,3,4], Edina Rosta[5,*], Walter Kolch[2,3,4,*], Nicolae-Viorel Buchete[1,*]

[1]*School of Physics & Complex and Adaptive Systems Laboratory, University College Dublin, Belfield, Dublin 4, Ireland*

[2]*Systems Biology Ireland, University College Dublin, Belfield, Dublin 4, Ireland*

[3]*Conway Institute, University College Dublin, Belfield, Dublin 4, Ireland*

[4]*School of Medicine, University College Dublin, Belfield, Dublin 4, Ireland*

[5]*Department of Chemistry, King's College London, SE1 1DB, United Kingdom*

Date: September 19th, 2016

Ω Equal contribution

Ψ Current address: *Department of Biochemistry and Molecular Biology, School of Biomedical Sciences and Biomedical Discovery Institute, Monash University, Melbourne, VIC 3800, Australia*

*E-mails: buchete@ucd.ie, edina.rosta@kcl.ac.uk, walter.kolch@ucd.ie

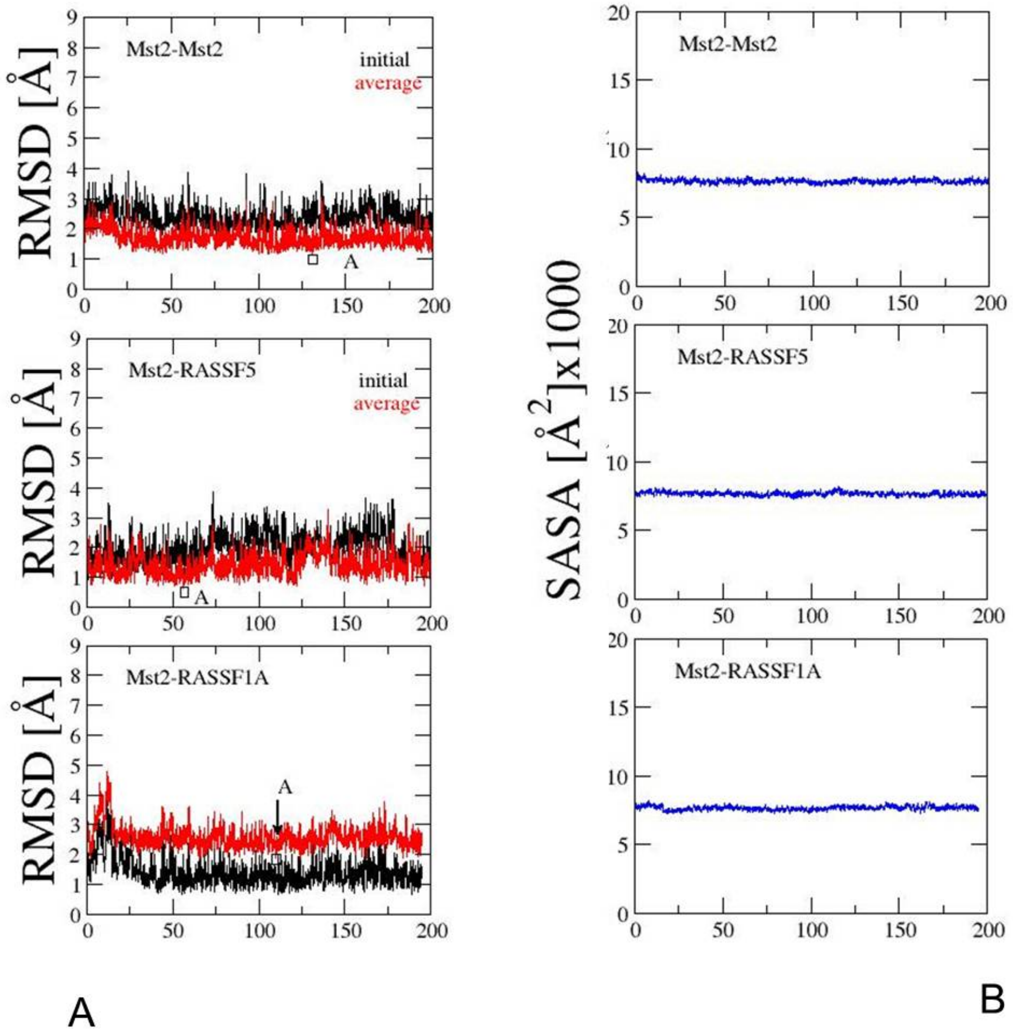

Figure S1. (A) C_α RMSD values for atomistic MD trajectories of representative structures of MST2 homo- and heterodimers, calculated with respect to the initial (black) and average (red) reference structures. Representative MD structures (i.e., smallest RMSD with respect to the average) are marked with small squares for MST2-MST2 (131.3 ns), MST2-RASSF5 (56.2 ns), and MST2-RASSF1A (109.9 ns). (B) The solvent accessible area is also stable for all these dimeric systems.

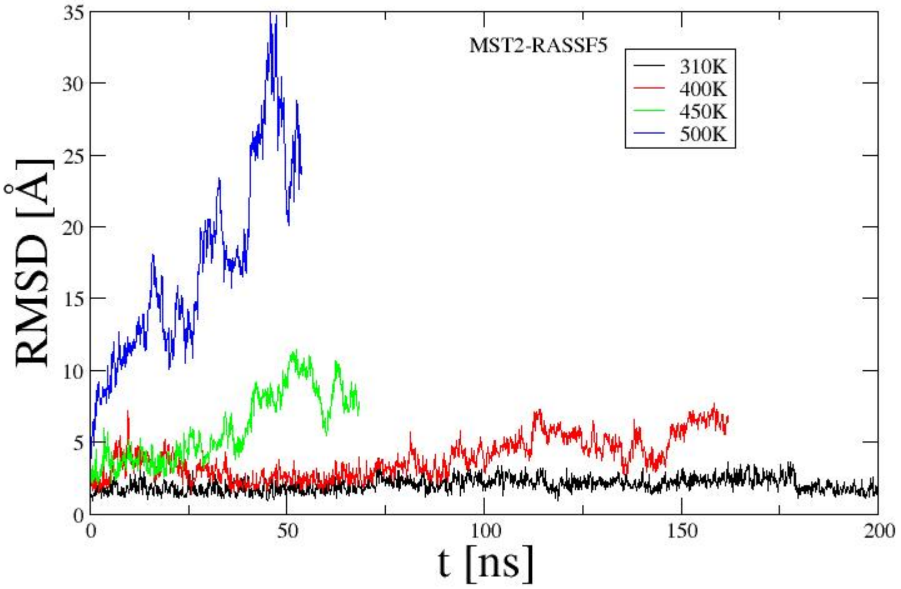

Figure S2. C_α RMSD values for atomistic MD trajectories of the representative MST2-RASSF5 dimer structure at 310 K (black), 400 K (red), 450 K (green), and 500 K (blue).

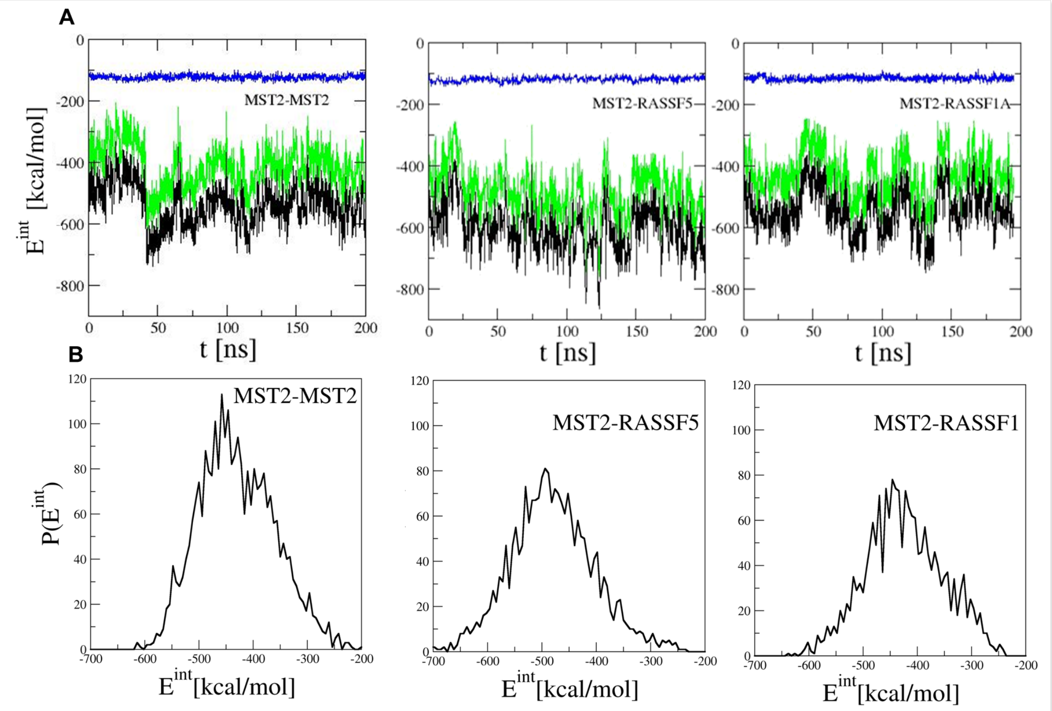

Figure S3. A) Total interaction energy (black line) between both protomers of MST2-MST2, MST2-RASSF5, and MST2-RASSF1A dimers, including electrostatic (green), and van der Waals (blue) contributions. **B)** Histograms of the total interaction energy between SARAH domain protomers for MST2-MST2, MST2-RASSF5, and MST2-RASSF1A systems.

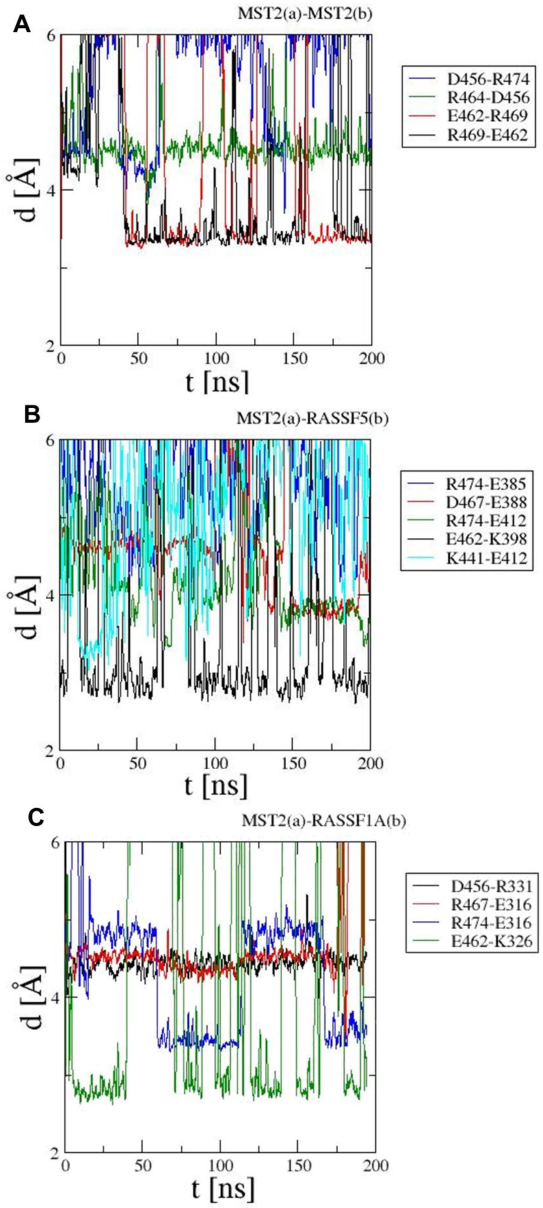

Figure S4. Distances corresponding to several important salt bridges along MD trajectories of MST2-MST2, MST2-RASSF5 and MST2-RASFF1A dimers.

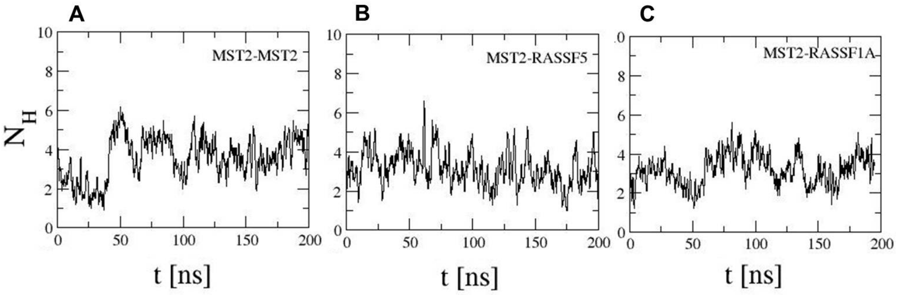

Figure S5. Number of hydrogen bonds between protomers along MD trajectories for MST2-MST2 (black), MST2-RASSF5 (blue), and MST2-RASSF1A (green) dimers.

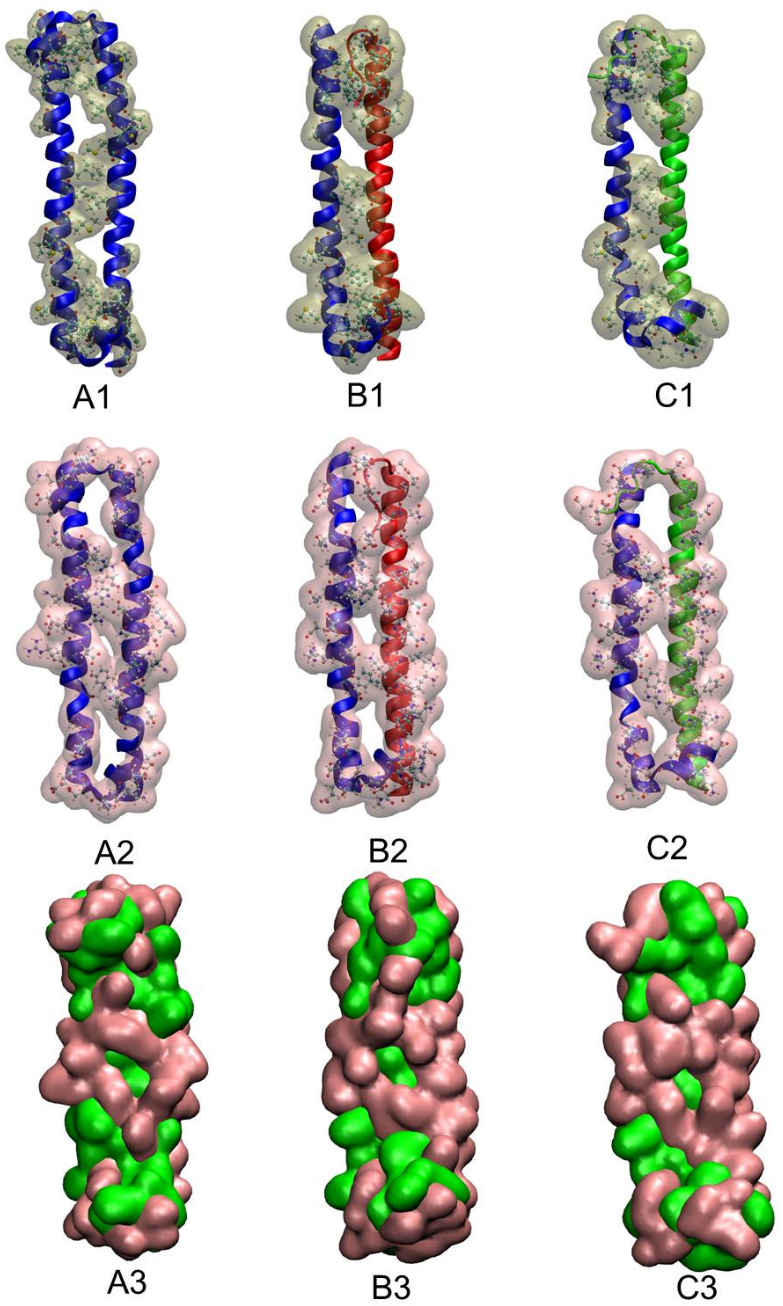

Figure S6. Hydrophobic and hydrophilic surfaces: (A) MST2-MST2 (blue-blue), (B) MST2-RASSF5 (blue-red), and (C) MST2-RASSF1A (blue-green). The A1-C1 pannels illustrate only the hydrophobic aminoacids (green), A2-C2 show the hydrophilic ones (pink), and A3-C3 show both the hydrophobic (green) and hydrophilic (pink) areas, respectively.

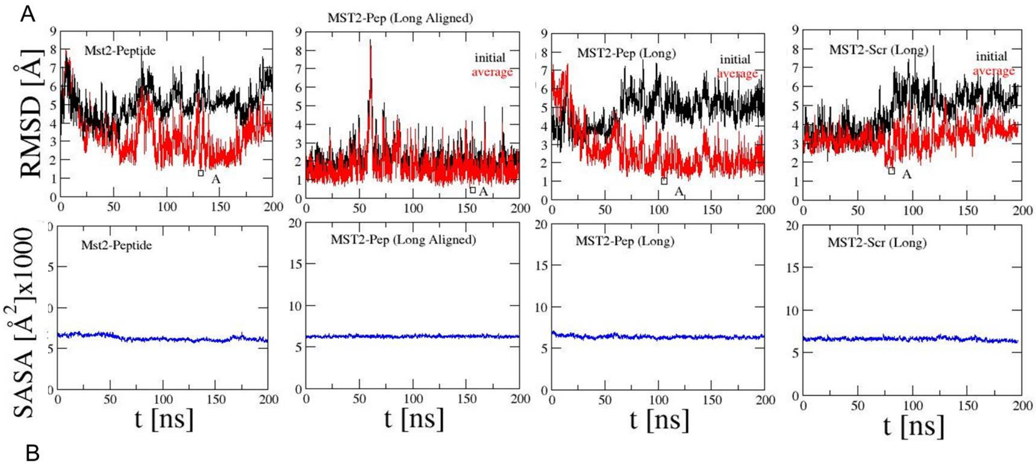

Figure S7. (A) C_α RMSD values for atomistic MD trajectories for the representative structures of MST2-Peptide heterodimers (see text for details). RMSD values were calculated with respect to the initial (black) and average (red) structures over the atomistic MD trajectories. Representative structures (i.e., smallest RMSD with respect to the average) are marked with small squares for MST2-PEP$_S$, MST2-PEP$_A$ MST2-PEP$_L$, and MST2-SCR. (B) The solvent accessible area is also remarkably stable for all four dimer systems.

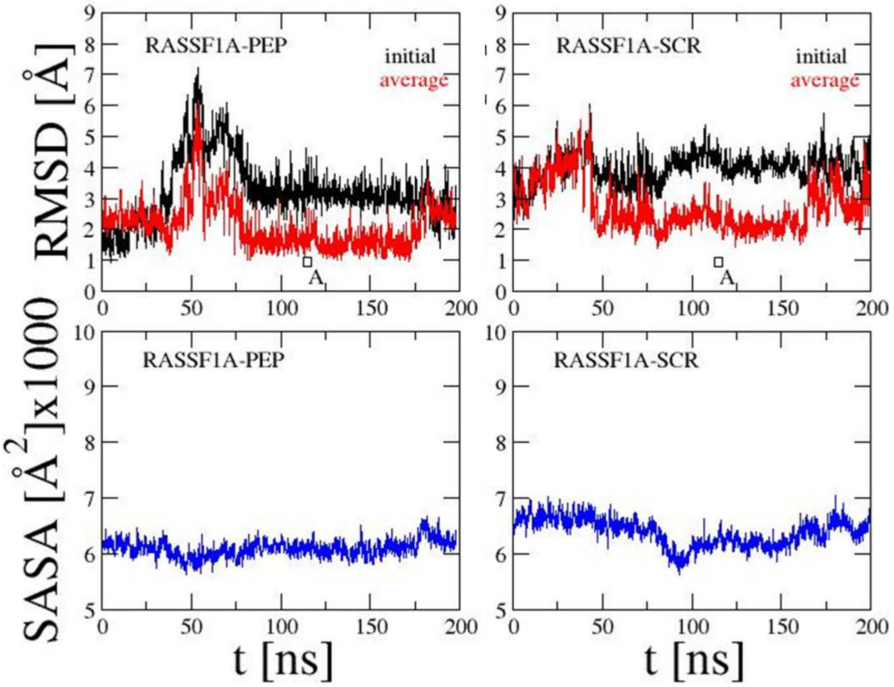

Figure S8. (A) C_α RMSD values for atomistic MD trajectories for structures of RASSF1A-Peptide heterodimers. RMSD values were calculated with respect to the initial (black) and average (red) structures. Representative structures (i.e., smallest RMSD with respect to the average) are marked with small squares for RASSF1A-PEP_A and RASSF1A-SCR. (B) The solvent accessible area is also remarkably stable.

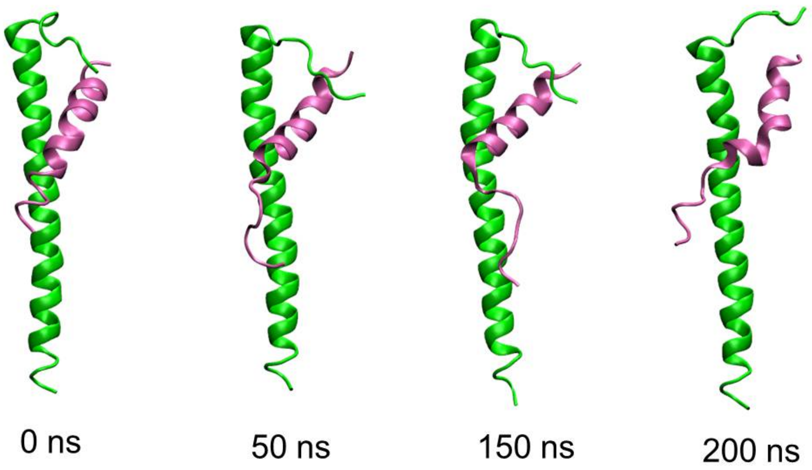

Figure S9. Sample structures of RASSF1A-SCR dimers from the atomistic MD trajectory at 0, 50, 150 and 200 ns. RASSF1A SARAH and the SCR ("scrambled" sequence) peptides are shown in green and pink, respectively. See text for details.

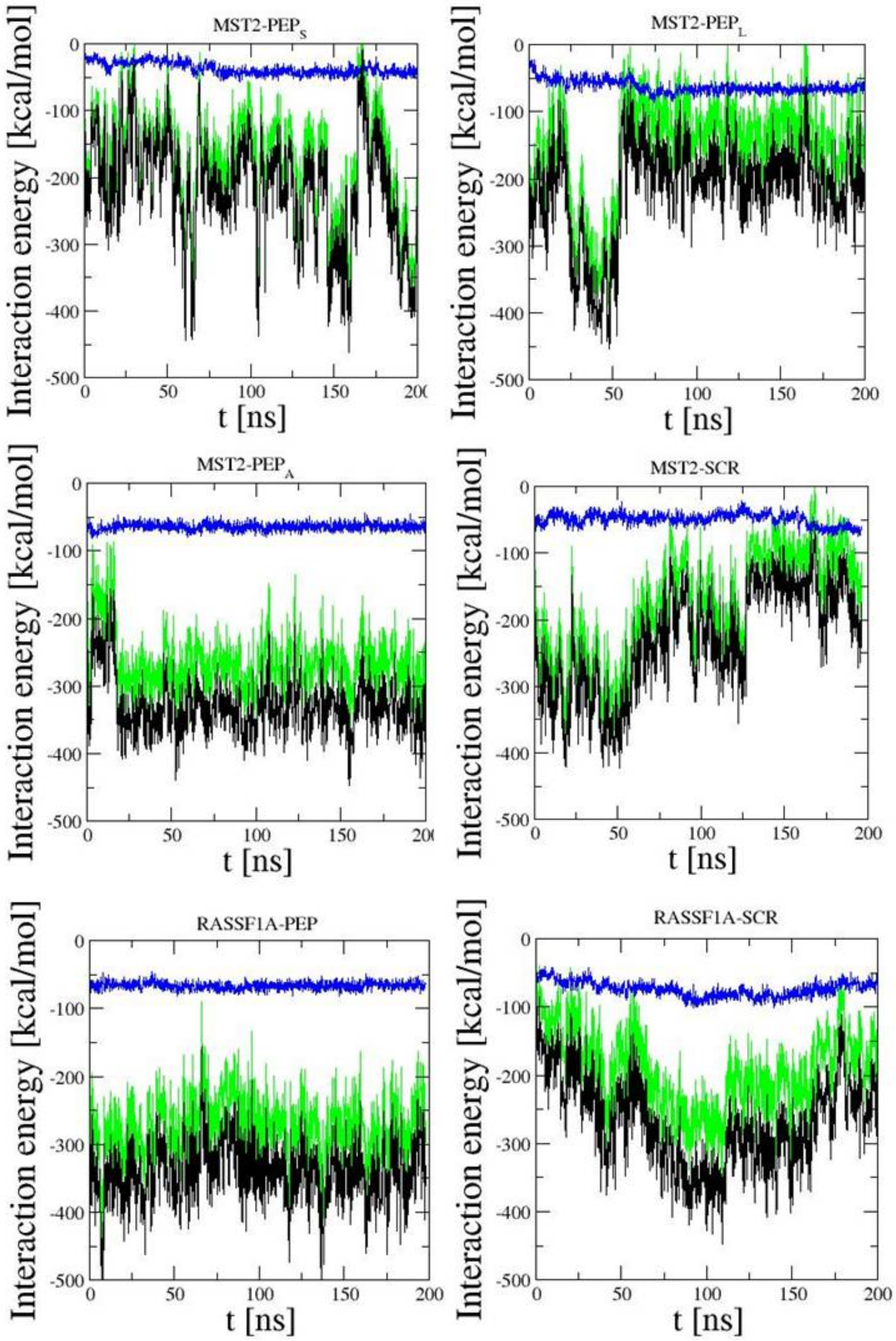

Figure S10. Total interaction energy (black) between both protomers of MST2-PEP$_S$, MST2-PEP$_L$, MST2-PEP$_A$, MST2-SCR, RASSF1A-PEP$_A$ and RASSF1-SCR dimers, including the electrostatic (green) and van der Waals (blue) energy contributions.

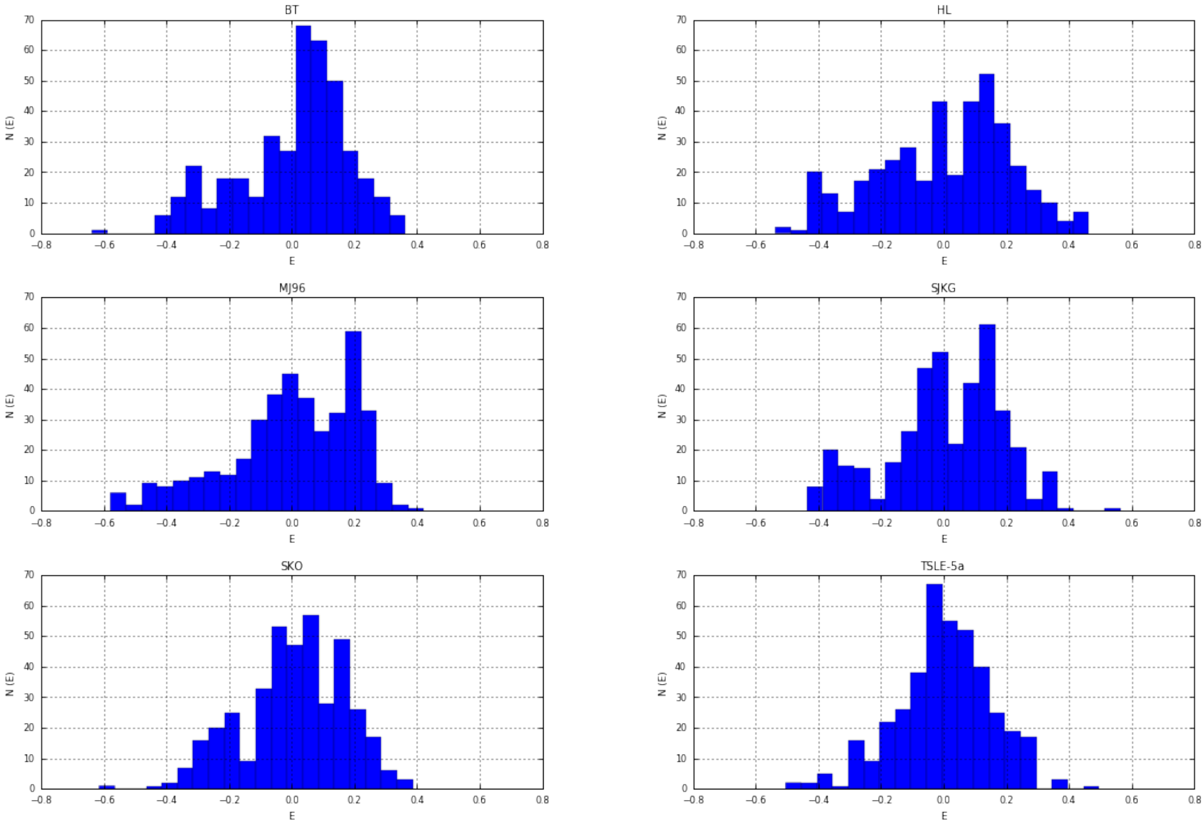

Figure S11. Comparison of histograms of residue-residue contact potential values from 20 x 20 contact potential matrices[1] developed by Hinds and Levitt (HL[2]), Betancourt and Thirumalai (BT[3]), Miyazawa and Jernigan (MJ-99[4]), Skolnick et al. (SJKG[5] , and SKO from Table 1a in Ref. [6]), and Tobi et al.[7] (TSLE from Table 5a in Ref.[7]).

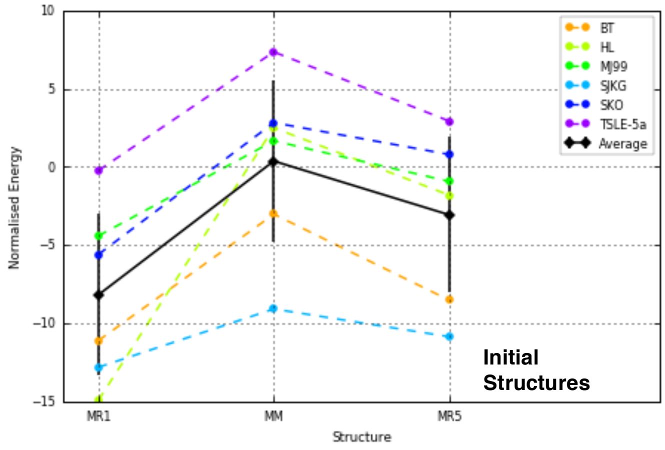

A

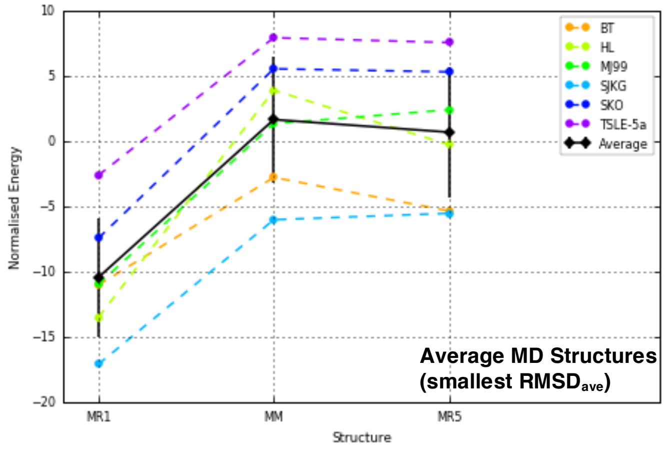

B

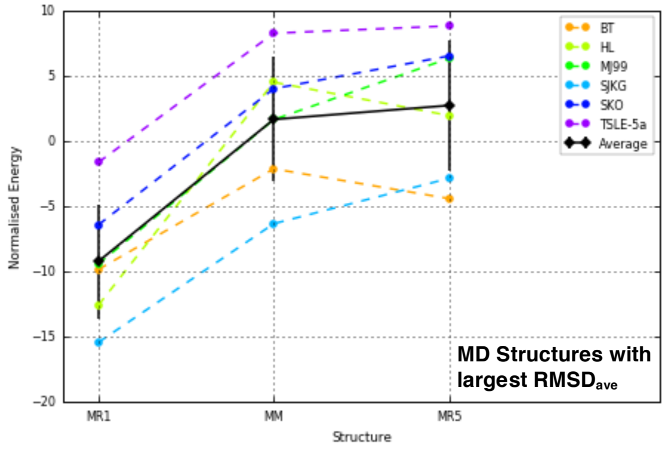

C

Figure S12. Contact interaction potential values calculated for structures of MST2-RASSF1 (notation MR1 on the horizontal axis), MST2-MST2 (MM) and MST2-RASSF5 (MR5) dimers. The calculations were performed for the six popular contact potentials represented in Fig. S11 for three cases: (A) dimer structures before MD, (B) the dimer structures from our MD simulations corresponding to frames with the smallest RMSD values compared to the average over the respective trajectory (RMSD$_{ave}$), and (C) the dimer structures from the same MD trajectories but corresponding to frames with the largest RMSD$_{ave}$ (to illustrate that even in this case the relative values for MR1 are still smaller than for MM

and MR5 dimers). A residue-residue contact cut-off distance of 5.5 Å between side-chain atoms was used. See text for details.

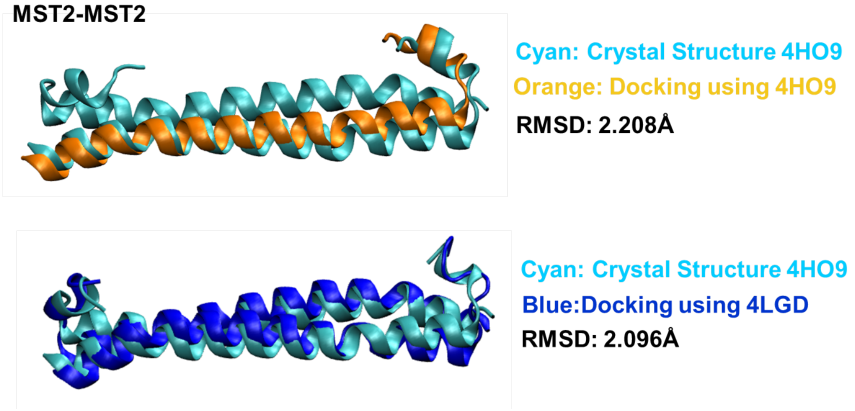

Figure S13. Up: In order to provide further information on the MST2-MST2 homodimer, we have carried out a docking study (yellow plot in the histogram in Fig. 3) of the MST2-MST2 dimer using the crystal structure available (4HO9). We have evaluated the structural differences between the 4HO9 structure and the results from docking using 4HO9 as template. Our results indicate that the differences between both docked and crystal structures are remarkably small, corresponding to alpha carbon (CA) RMSD of 2.208Å. **Down:** Furthermore, we have repeated the experiment, comparing the dimer obtained from docking using 4LGD (MST2 monomer) and the available 4HO9 crystal structure. Once more, the docking results are in very high agreement with the crystal structure, with a CA RMSD of 2.096 Å.

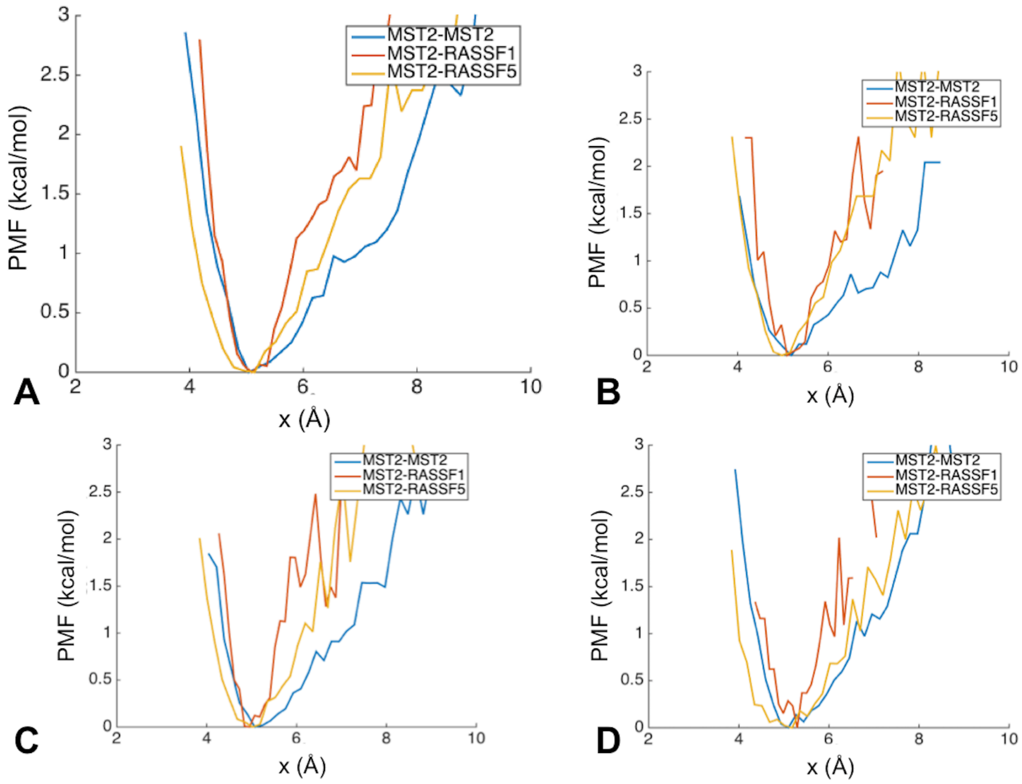

Figure S14. Potential of mean force (PMF) profiles for the MST2-MST2, MST2-RASSF1 and MST2-RASSF5 systems calculated from the corresponding dimer all-atom MD simulations using the recent dynamic histogram analysis method (DHAM) method.[8] Here x is the CA-CA distance between the two monomers that has the smallest average value along the corresponding MD trajectory. To probe convergence, the profiles were calculated for (A) the full trajectory, (B) the first third of the data, (C) the second third, and (D) the final third of the data. The first 20 ns (i.e., ~10%) of data from each trajectory were not included in this analysis.

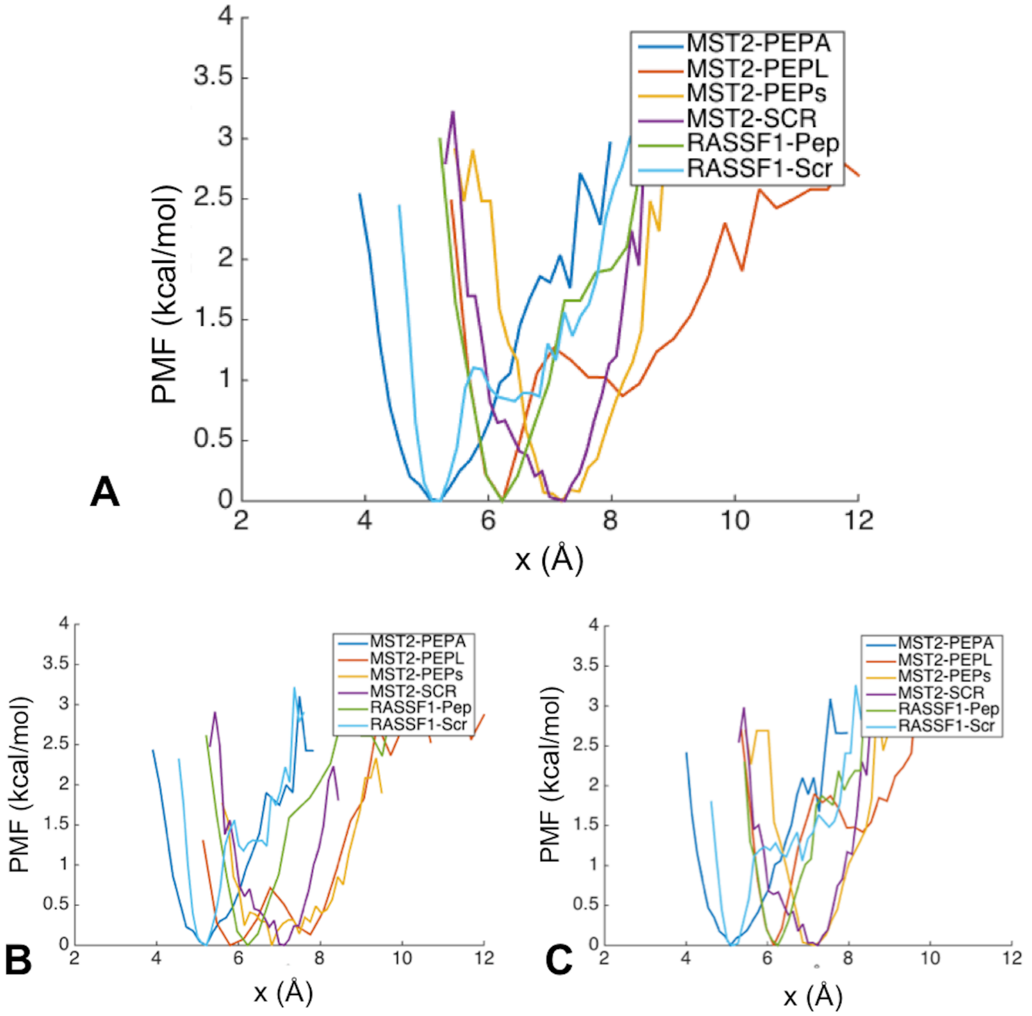

Figure S15. Potential of mean force (PMF) profiles for the MST2-PEPA, MST2-PEPL, MST2-PEPs, MST2-SCR, RASSF1-Pep, and RASSF1-Scr systems calculated from the corresponding dimer all-atom MD simulations using the recent dynamic histogram analysis method (DHAM) method.[8] Here *x* is the CA-CA distance between the two monomers that has the smallest average value along each corresponding MD trajectory. To probe convergence, the profiles were calculated for (A) the full trajectory, (B) the first half of the data, and (C) the second half of the data. The first 20 ns (i.e., ~10%) of data from each trajectory were not included in this analysis.

References

1. Buchete NV, Straub JE, Thirumalai D. Dissecting contact potentials for proteins: Relative contributions of individual amino acids. Proteins: Structure, Function, and Bioinformatics. 2008;70(1):119-30. doi: 10.1002/prot.21538.
2. Hinds DA, Levitt M. A lattice model for protein structure prediction at low resolution. Proceedings of the National Academy of Sciences of the United States of America. 1992;89(7):2536-40. PubMed PMID: PMC48696.
3. Betancourt MR, Thirumalai D. Pair potentials for protein folding: Choice of reference states and sensitivity of predicted native states to variations in the interaction schemes. Protein Science. 1999;8(2):361-9. doi: 10.1110/ps.8.2.361.
4. Miyazawa S, Jernigan RL. Self-consistent estimation of inter-residue protein contact energies based on an equilibrium mixture approximation of residues. Proteins: Structure, Function, and Bioinformatics. 1999;34(1):49-68. doi: 10.1002/(SICI)1097-0134(19990101)34:1<49::AID-PROT5>3.0.CO;2-L.
5. Skolnick J, Kolinski A, Ortiz A. Derivation of protein-specific pair potentials based on weak sequence fragment similarity. Proteins: Structure, Function, and Bioinformatics. 2000;38(1):3-16. doi: 10.1002/(SICI)1097-0134(20000101)38:1<3::AID-PROT2>3.0.CO;2-S.
6. Skolnick J, Godzik A, Jaroszewski L, Kolinski A. Derivation and testing of pair potentials for protein folding. When is the quasichemical approximation correct? Protein Science. 1997;6(3):676-88. doi: 10.1002/pro.5560060317.
7. Tobi D, Shafran G, Linial N, Elber R. On the design and analysis of protein folding potentials. Proteins: Structure, Function, and Bioinformatics. 2000;40(1):71-85. doi: 10.1002/(SICI)1097-0134(20000701)40:1<71::AID-PROT90>3.0.CO;2-3.
8. Rosta E, Hummer G. Free Energies from Dynamic Weighted Histogram Analysis Using Unbiased Markov State Model. Journal of Chemical Theory and Computation. 2015;11(1):276-85. doi: 10.1021/ct500719p.
